# Methodological Differences in Measuring Age Bias among Doctoral Psychology Trainees

**DOI:** 10.1093/geroni/igaf122.2208

**Published:** 2025-12-31

**Authors:** Benjamin Johnson, Grace Caskie

**Affiliations:** Lehigh University, Bethlehem, Pennsylvania, United States; Lehigh University, Bethlehem, Pennsylvania, United States

## Abstract

Nearly 90% of ageism measures utilize explicitly negative statements about older adults. Accurately measuring ageism with explicitly ageist statements may be hampered by social desirability bias, particularly among doctoral psychology trainees. To compare different methodological approaches to measuring ageism, 80 doctoral psychology trainees (23-33 years) completed explicit (FSA, ASD, AmbAS Hostile), implicit (Age IAT), and indirect (FAQ Negative Bias) ageism measures online, as well as a social desirability scale. Normalized to a 0-10 range for direct comparability, age bias was highest on the Age IAT (M = 2.70), followed by explicit measures (ASD, M = 2.67; FSA, M = 2.14; AmbAS Hostile, M = 2.10), and was least on the indirect measure (FAQ Negative Bias, M = 1.90). Follow-up analyses indicated the FSA Stereotypes subscale showed higher (M = 3.30) age bias than any full-scale score. Correlations of the Age IAT with explicit and indirect measures were non-significant. However, age bias measured indirectly (FAQ Negative Bias) correlated significantly with age bias measured explicitly (FSA total, FSA Stereotypes), even after partialling out social desirability; these correlations strengthened after controlling for social desirability. The indirect ageism measure overlapped more with explicit measures than the implicit measure and generally indicated less age bias, while explicit measures and the implicit measure, which focused on ageist stereotypes, produced higher age bias. Ageist stereotypes can operate unconsciously, which may pose harmful consequences in doctoral psychology trainees’ future work with older adult clients. Thus, different methodological approaches for measuring ageism influence the amount of self-reported ageism, suggesting that researchers and educators carefully consider their methodological approach when assessing ageism.

